# Patients' Contributions to Hospitals

**Published:** 1888-05-05

**Authors:** W. Burdett-Coutts


*(To be continued.)*

### PATIENTS' CONTRIBUTIONS TO HOSPITALS.

BY MR. W. BURDETT-COUTTS, M.P.

I MUST commence this paper by making my very grateful acknowledgments to the secretaries and other officers of the hospitals and dispensaries throughout Great Britain and Ireland who have done me the favour to send replies to the series of interrogatories addressed to them. In most cases where there was any information to give, it has been given freely and specifically to an extent which must have involved considerable trouble to those gentlemen, in addition to their ordinary work; and I not only thank them for their personal courtesy to myself, but I think I may assure them, on behalf of my audience to-night, that it will be taken as an evidence of the wider interest they, one and all, feel in the general advancement of the beneficent work in which they are engaged. Such an object alone could have justified me in putting them to so much trouble; and I claim that the special subject that I have chosen for this paper goes deep into the foundations of our hospital system.

PAY SYSTEM A RECOGNISED REMEDY.

In all fields of human thought and practice there is a natural tendency amongst individuals to concentrate their attention upon some particular point or defect; then to conceive some particular treatment of that point or remedy for that defect; next, to over-estimate the importance of the particular point in relation to the other component parts of the system, or to magnify the danger and incurability of the defect; and, lastly, to insensibly exaggerate the virtue and efficiency of the special remedy they have settled upon, until, by a process of mental nursing, somewhat similar to the development of the maternal instinct, and not unaccompanied by the vocal demonstrations common to the same, they come to believe and to assert that their offspring has assumed a perfectibility to which none other can pretend, and that it and it alone is born to save the tottering world into which they have projected it. This process may be called the manufacture of the "Fad." It is not unknown in the field of politics; it is one which occupies the time of a great many more or less prominent individuals in this country; and the results of it are to be found in so many isolated and uncongenial atoms floating restlessly about in the political and social systems, shouldering each other hither and thither, and alternately rising and falling on the surface of public attention, but serving no good or sound or justly-proportioned purpose of permanent and thorough reform.

From any such category I claim exemption for the Pay System—a system which obtains in every other civilised country, and is to be found working in at least two great classes of hospitals in our own, and which, both abroad and wherever it is adopted at home, can be shown to have secured a financial soundness which most of the great hospitals not adopting the system are proportionately deficient in, cannot by any stretch of prejudice be called a "fad."

FINANCIAL CONDITION OF HOSPITALS—THE OBJECT OF THIS PAPER.

I have claimed that this subject goes to the foundations of the system, because it is closely bound up with that very question of financial soundness which has now become a burning one with respect at least to the great Hospitals of London. And here I wish very earnestly to disavow to the professional part of my audience any intention of intruding within the limits of their especial sphere. There are questions to which the Pay System gives rise, but which, because they lie especially within the professional sphere, I shall not discuss. *Ne sutor ultra crepidam*, and my starting-point to-night is not within but outside of Hospitals. It has to do with what comes to them from without, and the only right which I can claim is to speak from the point of view of those who contribute to Hospitals.

Philanthropic Contributions.

I am not altogether without experience of the conditions and methods and objects of what I will call the eleemosynary principle, more abundant and far-reaching in this country than in any other, and I trust that I shall at least be credited with the intention of saying nothing which shall restrict, but rather of doing what I can to confirm and enlarge the operation of that principle with respect to the hospitals of this country.

The contributions made by the charitable to the London Hospitals amount to an enormous sum. They represent the sympathy with human suffering which is the primitive source and the traditional mainspring of charity, but alongside of which there has grown up in these days a very formidable competitor, the sympathy with human effort. Now, and more and more every day, charity can flow into ever-multiplying channels leading directly to moral, social, and intellectual improvement; it is summoned to the aid of numberless schemes for the advancement of the material interests of the poor; and while all these—although it may seem hard to say it—have more hope and promise of a fruitful future than the mere relief of suffering, in addition they, one and all, bear the legend which has become in our day such a potent factor in the eliciting and the organisation of benevolence, "Help people to help themselves." The charity which calls forth a responsive effort on the part of its object is fast winning the field in the latter quarter of the nineteenth century.

Insufficient to their Support.

The great free hospitals of London are splendid monuments of philanthropy. Far be it from me to touch them and their system in this paper, if you think that they are in a satisfactory or safe position. If they can go on as they are, I will gladly count this paper, and the personal labour it has involved, as dust and ashes. But if you find this enormous annual contribution from the charitable, stimulated as it is by every kind of ingenious appeal and urgent entreaty, unable, even with the aid of the great resources derived from funded property, to meet the demands of the system; if you find growing up amongst the contributing classes a feeling of hopelessness to cope with the demand, which will tend more than anything else to discourage subscribers; if you find deficits every year increasing, beds diminishing in number, the pressure of patients greater, the power of relief less; if, in short, you find the efficiency and stability of these great institutions seriously threatened; then, surely, it is not enough to stand gazing at them in fixed admiration, or pointing to them as monuments of this or that—you must put your finger on the weak spot and consider the remedy to apply.

Present and Future Financial Condition Unsound.

It is not enough to say that the present financial condition of London hospitals is unsatisfactory. It is worse than that, it is either bankrupt or subject to intermittent attacks of bankruptcy which from time to time are staved off by heroic remedies—but remedies which, the more you apply them, the more exhausted and less available do they become. "Bankrupt" is a big word, but it is not too big when confined within these limits—that the system, if it goes on as it is now, without any new and strong element of support being brought into it, cannot exist much longer as a system equal to supplying the hospital wants of London. It is in the fatal position in which increasing demand is met by shrinkage of supply. There seems to be no term or limit to the growth of London—in itself almost a nation—acquiring fresh territories every year and begetting new populations to inhabit them. The yearly increase has been reckoned at seventy thousand souls, and this ceaseless extension is a subject of grave and deep anxiety to all who feel any responsibility for the welfare of the countless thousands who are yet to come. It is this fact which warns us that it is not enough to live in the present, and from hand to mouth, but that we are trustees of the future; it is in that capacity and from that point of view that we are in duty bound to apply ourselves to every problem connected with the life of London. In many departments this obligation has been recognized, of which I might quote an obvious example—the provision and preservation of open spaces. Not even the present heavy burdens of the ratepayers have been able to turn their eyes from the incalculable importance of this movement to the future. Is it not well that we should take up the same attitude of prevision with regard to the great system of hospital relief, upon which the health and safety of this vast metropolis so largely depends. We may struggle on as we are for a few months or years, but our capacity is non-elastic, while the body of our work swells every day.

Deficit.

For the year 1886—I take the figures from a recent number of The Hospital—the total deficit of eighty-five of the chief general and special hospitals in London was £101,736, the expenses were £494,351, the income £392,615. I include dividends from funded property, but purposely leave legacies out of account.

Legacies not Reliable Source of Income.

It may be said that legacies, averaged over a series of years, afford a reliable source of income. But is it so? They come from the dead, As long as they come, well and good; but if they fail, their contributors are beyond the reach of those appeals which when made to the living and for great emergencies—for great institutions which are on the brink of ruin—have always a possibility of success. The precarious nature of this source of income is illustrated by the fact that, in the year previous to that under consideration, there was a deficit of £80,000 after taking credit for legacies. At least it would be wise to consider legacies as windfalls, to be gratefully added to the system when we have given it some principle of inherent self-support. I believe such a principle is to be found in the pay system. As to its more immediate results in London, we have seen that 25 per cent. more than the present income is required to make up the deficiency. I am of opinion that if a proper pay system were adopted in both the out-patient and in-patients departments of these hospitals, this deficiency would be made up by the payments of patients. I propose in the course of this paper to adduce reasons for this belief.

Effects on the Public Mind.

So much for the hospital side of the question. Now for my side of it. Speaking from our point of view, we and many other persons who form what I might call targets for every institution to shoot its arrow at, have become simply appalled by the demands made by the London hospitals. I can assure you that no willingness to strain every nerve to meet it can compete with the increasing and pressing demand. The position is most discouraging, and this in spite of the existence in spirit and in practice of a magnificent system of philanthropy which is the envy and the admiration of the rest of the world. From this point of view, if from no other, I feel that when we have this splendid instrument of philanthropy in our hands it is our bounden duty to see that it is not weakened and discouraged, and we shall best secure this object by providing that it shall be used with a close consideration to its efficiency, used so as to secure the best results, used solely for the objects for which it is given, and used upon those objects in a manner and to an extent so justly proportioned to their necessities that each contribution, however small, shall be made to command the greatest possible relief.

THE BLOT IN THE SYSTEM, A FALSE ECONOMIC BASIS.

I have now arrived at the blot in the system. Of charity there is enough, but under the present system, what I may call the incidence of charity is wrong; that is to say, those who can help themselves, wholly or in part, are helped entirely from charity, and those who cannot help themselves therefore are pushed to a certain extent into the cold.

No amount of charity that can be called forth in this country or in any other will ever suffice to supply any particular want to those who can afford to supply it to themselves. This general canon applies both to assistance in whole or in part; for, in proportion to the accuracy with which you adjust the incidence of charity, in that proportion you multiply the cases to which that charity will give efficient help, enlarge the area over which it extends, and increase the aggregate of good done by the aggregate of money given. Once assure contributors that you are really doing this, and there will be no lack of contributors. The day of promiscuous charity is past, or at least all intelligent people are striving that the utmost results shall be obtained from the money given.

PROMISCUOUS CHARITY.

But the present is a system of promiscuous charity, and therefore I say that the hospitals which adopt it are on a false economic basis. I am no stickler for theoretical perfectibility. I repeat, if you think you can go on as you are doing, I wish you God-speed and a good deliverance from the straits to which you cannot shut your eyes; but I venture to warn you that sooner or later the contributor will have his eyes opened to the fact that, with all the labour and patience and self-sacrifice and heroism and devotion to duty which characterises one of the noblest professions, you are not doing justice to his objects and intentions, you are not devoting his money where and how it is most needed, and if such an idea once becomes prevalent in the public mind, a frost will settle on your charitable income which it will take years to thaw.

RESTRICTS ITS REAL AREA.

Let no man say I am arguing against the poor. I am arguing for them, for the admission of a greater number of them to the great benefits of our hospitals. I desire, and I shall always strive, that every absolutely poor person shall be treated free of cost during illness; that every other person, with respect to such portion of the cost of such treatment as he is unable to pay, shall to that extent be treated free of cost, and if you can find a system which will enable you to approach, even within measurable distance of such an adjustment as this, you will not only in my humble opinion increase and foster the charitable contributions, but you will solve the financial difficulties of your hospitals.

